# Combining Non-Uniform Time Slice and Finite Difference to Improve 3D Ghost Imaging

**DOI:** 10.3390/s19020418

**Published:** 2019-01-21

**Authors:** Fanghua Zhang, Jie Cao, Qun Hao, Kaiyu Zhang, Yang Cheng, Yingbo Wang, Yongchao Feng

**Affiliations:** 1 School of optics and photonics, Beijing Institute of Technology, Key Laboratory of Biomimetic Robots and Systems, Ministry of Education, Beijing 100081, China; zfh135@163.com (F.Z.); dxzky@126.com (K.Z.); yangcheng2007@163.com (Y.C.); wangyb_0309@163.com (Y.W.); fych1015@163.com (Y.F.); 2 NUS Suzhou Research Institute (NUSRI), Suzhou Industrial Park, Suzhou 215123, China

**Keywords:** three-dimensional ghost imaging, differential optical path, non-uniform time slice, computational imaging

## Abstract

Three-dimensional ghost imaging (3DGI) using a detector is widely used in many applications. The performance of 3DGI based on a uniform time slice is difficult to improve because obtaining an accurate time-slice position remains a challenge. This paper reports a novel structure based on non-uniform time slice combined with finite difference. In this approach, finite difference is beneficial to improving sensitivity of zero crossing to accurately obtain the position of the target in the field of view. Simultaneously, non-uniform time slice is used to quickly obtain 3DGI on an interesting target. Results show that better performances of 3DGI are obtained by our proposed method compared to the traditional method. Moreover, the relation between time slice and the signal-noise-ratio of 3DGI is discussed, and the optimal differential distance is obtained, thus motivating the development of a high-performance 3DGI.

## 1. Introduction

Three-dimensional (3D) light detection and ranging (lidar) based on the principle of time of flight (TOF) is widely used in various fields, such as remote sensing [1], machine vision [2], and target detection and recognition [3], because of its active long-range detection and high resolution. Traditional 3D lidar methods are classified into scanning and non-scanning types [4]. Compared with TOF-based lidar, ghost imaging (GI) lidar, as a novel approach based on a single detector, is noteworthy because of its high performances, including speed, detection efficiency [5], no Rayleigh limitation [6], and signal-to-noise ratio (SNR) [7]. Compared with traditional scanning TOF-based lidar, GI lidar has easily recognizable compacted size and high speed [8] because GI does not need any scanning devices. Additionally, scanning TOF-based lidar is limited by the Rayleigh limitation of the emitting lens aperture. GI lidar also obtains the target image even without a lens [9]. Compared with non-scanning TOF-based lidar, such as that based on an avalanche photodiode (APD) array, GI lidar obtains higher detecting sensitivity because echo signal power from the target focusing on a single APD is stronger than on an APD array illuminated by floodlight illumination [10,11].Therefore, GI lidar obtains higher SNR than non-scanning TOF-based lidar [12]. 

In early GI research, researchers mainly focused on two-dimensional GI (2DGI) [13]. With the development of light source and computational imaging, 3DGI has gained the interest of researchers because it provides numerous target details. For example, four single-pixel detectors in different locations are employed to construct a 3D computational image [14]. In this experiment, a 2D image is obtained by each single-pixel detector, and then a 3D image is reconstructed by combining the shading information of surface gradient in each 2D image. However, the computational process to reconstruct a 3D image is time consuming [15]. Conversely, the time-slice method is used to construct 3DGI [16,17,18], which can overcome the backscatter brought by obscurants, camouflage, and water. Therefore, 3DGI based on this method is more suitable for long-range detection. A method was proposed using this structure. Some studies have investigated this method based on imaging efficiency [8,19], accuracy [17], and resolution [18] through simulations and experiments. However, a common feature of 3DGI in previous studies was their basis on the uniform time-slice method (UTSM), which results in low efficiency and unfavorable performances in the reconstruction of 3D imaging. In most situations, however, we merely want to clearly observe the interesting part rather than the whole field of view (FOV), e.g., target recognition or tracking, so a non-uniform sampling method has been studied in a space domain [20,21] in 2D imaging and has obtained a favorable performance. Different from 2D imaging, the depth information of 3DGI lidar is based on each time-slice position on an echo signal, which are full-waveform signal from scenes, and the accuracy of the position of the time slice on echo signal is important to improve the 3DGI performance [17]. Although previous studies on 3DGI applied a non-uniform time-slice method (NUTSM), to the best of our knowledge, the relationship between the accuracy of time slice and the performance of 3DGI has not been studied according to the target of interest [22]. Different from reference [22], we not only study the relationship between the position of time slice and GI, but also report a finite difference instead of peak detection to improve accuracy of time slice for the situation of imaging with high resolution and real time, e.g., target tracking. NUTSM is more suitable than UTSM because of larger redundant data in the latter. Meanwhile, UNTSM is based on the peak of echo signal, which is beneficial in order to obtain the salient feature in the scene [23], especially for a large depth field of view, because UTSM with high resolution results in too large data to reconstruction for high efficiency. Therefore, on the basis of the echo signal detected by a single-pixel detector, we proposed a NUTSM combined with finite differential in time domain to improve the performance of 3DGI. The rest of the paper is organized as follows. The method of NUTSM with finite difference is studied in Section 2. Based on that, modelling verification and comparative results are obtained through systematic simulations in Section 3. The optimal parameter and related issue on NUTSM are discussed in Section 4. 

## 2. Method

### 2.1. Combining NUTSM with Finite Difference 

**Figure 1:**
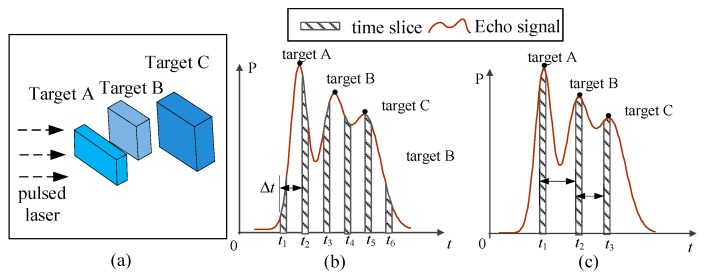
Comparison between uniform and non-uniform time-slice sequences. (**a**) Multiple targets are illuminated by pulsed laser. (**b**) Traditional uniform time-slice method (UTSM), and the interval between neighbor time slices is uniform. (**c**) Non-uniform time-slice method (NUTSM), and time slice are located on peak positions.

Figure 1 shows the difference between UTSM and NUTSM. Figure 1a shows multiple targets (A, B, and C) in the scene. The feature of UTSM has the same time interval (*△t*) between adjacent sampling units (Figure 1b). Such a method results in numerous calculations in real-time tasks because high-resolution GI requires large acquisition data. Meanwhile, in NUTSM, the time intervals between adjacent sampling units are different (Figure 1c). Specifically, the position of time slices is determined by the peaks in echo signal. However, the peak position is easily affected by pulse broadening and pulse distortion under long-range detection [24]. Hence, accurately extracting the peak position becomes difficult and results in a low accuracy of 3DGI for the targets of interest.

**Figure 2:**
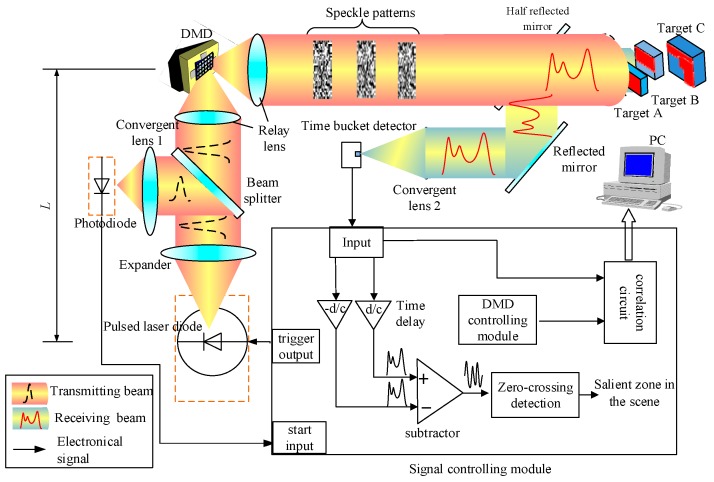
Principle of 3D ghost imaging (GI) based on non-uniform time-slice method (NUTSM) combined with finite difference.

**Figure 3:**
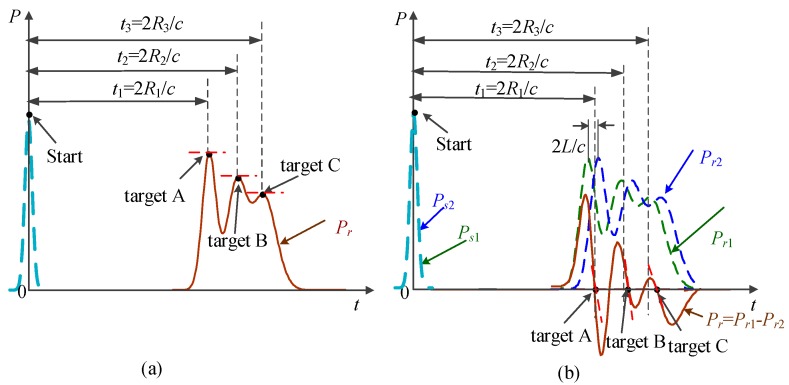
Echo signal comparison between peak and zero crossing. (**a**) Peak position by single time-resolved bucket detector. (**b**) Zero crossing by finite difference.

The principle of 3DGI based on NUTSM combined with finite difference method is shown in Figure 2. The pulsed laser diode is triggered by a signal-controlling module. The transmitting beam goes through an expander and is divided into two parts. One part is used to produce a start signal by using a fast photodiode. The other part is an illuminating digital micromirror device (DMD) that produces random speckles to illuminate the target through a half-reflective mirror. Then, the reflected or scattered beams from the targets travel to the half-reflective mirror, a reflective mirror, and a convergent lens, and illuminate time-resolved bucket detectors. The echo signals are delayed (*d*/c) and (−*d*/c), where d differential distance, and then it is subtracted between each other to produce zero crossing in the time domain (Figure 3b). Meanwhile, the signals from the time-resolved bucket detector used to correlate with the speckle patterns produced by DMD. To obtain 3DGI, we use a time-resolved bucket detector to receive the intensity of the target at the different time slices. In the system, the speckle patterns, such as the reference arm, can be pre-computed by controlling the DMD. On the basis of Figure 2, the three targets are reflected by peaks in echo signals (Figure 3a) but such a position is difficult to extract when the pulse width is broadened [25], specifically for long distance due to background noise [26]. Meanwhile, the accuracy of the extracting peak becomes poor because of the peak’s low sensitivity, i.e., the low gradient of the peak, shown in the red dot-dash line (Figure 3a). Therefore, we introduce the finite difference method to transfer the peak into a zero crossing (Figure 3b). The effects from the pulse broadening and low sensitivity are thus mitigated remarkably [27]. 

### 2.2. Theory

**Figure 4:**
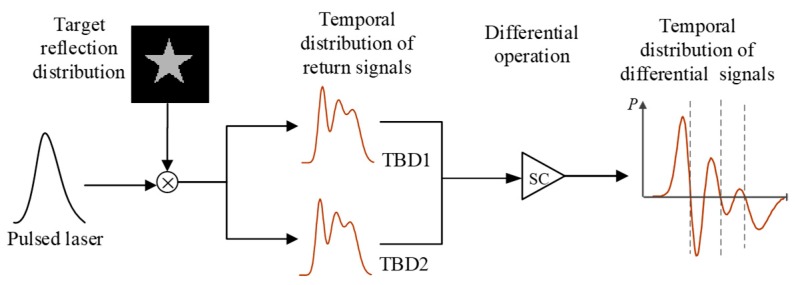
Step I: Depth information of targets is extracted based on finite difference.

The proposed method includes two steps as follows: 

Step I: The depth information of the targets is extracted to determine the number of targets in FOV (Figure 4). Thus, all the elements of DMD are in “ON” status during the process, that is, DMD is viewed as a mirror. After this step, the accurate position of each target is obtained and then used to produce non-uniform time slices in the next step. 

**Figure 5:**
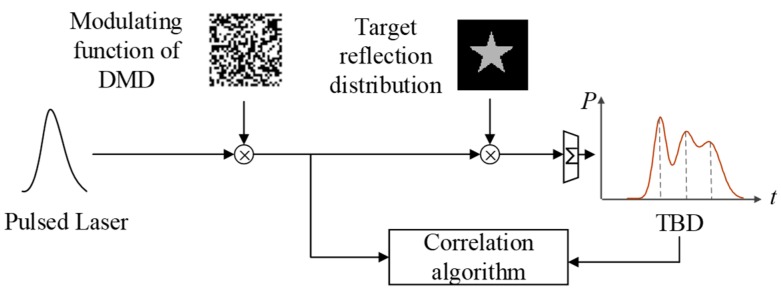
Step II: 3DGI is constructed by using non-uniform time slices.

Step II: 3DGI is constructed. Based on step I, the random speckle patterns are produced by DMD, and the 2DGI is obtained by correlating the light field and the intensity of the time-resolved bucket detector. Therefore, 3DGI is constructed by combining 2DGI according to the depths of non-uniform time slices, shown in Figure 5.

The pulsed laser is approximately viewed as a time-space domain Gaussian function, which is written as [28]

(1) $$\left\{ \begin{array}{l} P\left(x,y,z,t\right)=\frac{P_{0}}{\tau \sqrt{2\pi }}\exp\left[-\frac{{\left(t-z/c\right)}^{2}}{2\tau^{2}}\right]\cdot \frac{1}{\pi W{\left(z\right)}^{2}}\exp\left[-\frac{x^{2}+y^{2}}{W{\left(z\right)}^{2}}\right]\\ W\left(z\right)=W_{0}\left[1+{\left(\frac{\lambda z}{\pi W_{0}}\right)}^{2}\right] \end{array}\right.$$

where *P*_0_ is the pulse energy, *τ* is the transmitting pulse width, *W*_0_ is the waist radius of the laser source, *W(z)* is the beam radius at the distance of z from the laser source, *c* is the light speed, *λ* is the wavelength, and *x* and *y* are the coordinates of the *z* plane. The pulsed laser illuminates the targets in FOV, and the intensity of the echo signal is expressed as

(2) $$\left\{ \begin{array}{l} P_{r}\left(t\right)=\sum_{n=1}^{N}\iint \frac{T_{a}^{2}T_{0}P_{0}\eta_{D}}{\sqrt{2\pi }\tau_{rn}}\exp\left[-\frac{1}{2\tau_{rn}^{2}}{\left(t-\frac{2R_{n}}{c}\right)}^{2}\right]\cdot \frac{1}{\pi W{\left(R_{n}\right)}^{2}}\cdot \exp\left[-\frac{x^{2}+y^{2}}{W{\left(R_{n}\right)}^{2}}\right]\rho_{rn}\left(x,y\right)\cos\varphi dxdy+P_{B}\\ \varphi =arc\tan\left(\frac{\sqrt{x^{2}+y^{2}}}{R_{n}}\right)\\ P_{B}=\iint \rho_{rn}\left(x,y\right)h_{sun}T_{0}A_{r}\sin{\left(\alpha /2\right)}^{2}\Delta \lambda dxdy\\ \tau_{rn}=\tau +\frac{\tan^{2}\left(\theta_{n}\right)W{\left(R_{n}\right)}^{2}}{c^{2}} \end{array}\right.$$

where *N* is the total number of targets in the FOV illuminated by the pulsed laser, *T_a_* is the one-way atmospheric transmission, *T_o_* is the optical efficiency, *R_n_* is the distance between the optical system and the *n*-th target, *c* is the light speed, *η_D_* is the quantum efficiency, *ρ_rn_ (x,y)* is the reflectivity with respect to the coordinate (*x,y*) of the *n*-th target plane, *φ* is the solid angle between the target point and detector, *W(R_n_)* is the beam radius of the *n*-th target plane at the distance of *Rn*, *P_B_* is the intensity of the background [29], *h_sun_* is the background solar irradiance, *A_r_* is the area of the receiver, *α* is the FOV of system, *△λ* is the optical bandwidth, and *τ_r_* is the receiving pulse width, which is related to the angle of the target [25,30]. Given that the depth information is merely determined by the echo signal of the time domain, let

(3) $$\Psi_{n}=\iint \frac{1}{\pi W{\left(R_{n}\right)}^{2}}\exp\left[-\frac{x^{2}+y^{2}}{W{\left(R_{n}\right)}^{2}}\right]\rho_{rn}\left(x,y\right)dxdy$$

where Ψ*_n_* belongs to the space domain, and *P_r_(t)* is transformed into

(4) $$P_{r}\left(t\right)=\sum_{n=1}^{N}\frac{\Psi_{n}T_{a}^{2}T_{0}P_{0}\eta_{D}}{\sqrt{2\pi }\tau_{r}}\exp\left[-\frac{1}{2\tau_{r}^{2}}{\left(t-\frac{2R_{n}}{c}\right)}^{2}\right]+P_{B}$$

According to Equation (2), the intensity of the differential echo signal is written as

(5) $$P_{rd}\left(t\right)=\sum_{n=1}^{N}\frac{\Psi_{n}T_{a}^{2}T_{0}P_{0}\eta_{D}}{\sqrt{2\pi }\tau_{r}}\left\{\exp\left[-\frac{1}{2\tau_{r}^{2}}{\left(t-\frac{2R_{n}-d}{c}\right)}^{2}\right]-\exp\left[-\frac{1}{2\tau_{r}^{2}}{\left(t-\frac{2R_{n}+d}{c}\right)}^{2}\right]\right\}$$

where *d* is the differential distance (Figure 2). From Equation (5), we find that the effects from the background are suppressed effectively, and the peak is transformed into a zero crossing (Figure 3b), which is beneficial for obtaining a considerably higher sensitivity than that of the peak. Therefore, depth information is extracted accurately by using the position of the zero crossing. After Step I, the time slice is determined by the depth information of each target in FOV. 

As shown in Figure 2c, multiple zero crossings mean multiple targets in FOV. The depth information of each target are obtained based on finite difference (i.e., *t_n_* (n = 1, 2,…, N)). Therefore, the GI based on each time slice of each target is constructed by using the correlation between the random light field from DMD and the intensity of the time-resolved bucket detector and is written as

(6) $$G_{n}\left(x,y\right)=\langle P_{1n}\left(x,y\right)\cdot P_{2n}\rangle -\langle P_{1n}\left(x,y\right)\rangle \langle P_{2n}\rangle$$

where *P*_1*n*_ is the intensity distribution of light field for the *n*-th time slice (i.e., *n*-th target), P_2*n*_ is the corresponding intensity of time-resolved bucket detector with respect to the *n*-th time slice, and < . > is the ensemble average. To simplify the theory, the working frequency of DMD is assumed to be the same as the laser source, and the laser intensity after modulating by DMD is written as

(7) $$P_{DMD}\left(x,y,t\right)=\frac{P_{0}}{\tau \sqrt{2\pi }}\cdot \exp\left[-\frac{{\left(t-L/c\right)}^{2}}{2\tau^{2}}\right]\cdot \frac{1}{\pi W{\left(L\right)}^{2}}\cdot \exp\left[-\frac{x^{2}+y^{2}}{W{\left(L\right)}^{2}}\right]\cdot I\left(x,y\right)$$

where *L* is the distance between the laser and the DMD, and *I*(*x,y*) is the modulated function of DMD. The depth information of each target is obtained by step I, that is, *t_n_* is determined after Step I. Therefore, we obtain the following:

(8) $$P_{1n}\left(x,y,t\right)=\frac{T_{a}P_{0}}{\tau \sqrt{2\pi }}\cdot \exp\left[-\frac{{\left(t-t_{n}\right)}^{2}}{2\tau^{2}}\right]\cdot \frac{1}{\pi W{\left(R_{n}\right)}^{2}}\cdot \exp\left[-\frac{x^{2}+y^{2}}{W{\left(R_{n}\right)}^{2}}\right]\cdot I\left(\frac{W\left(L\right)}{W\left(R_{n}\right)}x,\frac{W\left(L\right)}{W\left(R_{n}\right)}y\right)$$

The distribution of reflective intensity from the *n*-th target is written as

(9) $$P_{rn}\left(x,y,t\right)=P_{1n}\left(x,y,t\right)\cdot \rho_{rn}\left(x,y\right)$$

Therefore, the intensity of the echo signal from a single time-resolved bucket detector is written as

(10) $$P_{2n}\left(t\right)=\frac{T_{a}{}^{2}T_{0}P_{0}}{\tau_{r}\sqrt{2\pi }}\cdot \exp\left[-\frac{{\left(t-t_{n}\right)}^{2}}{2\tau^{2}}\right]\cdot \frac{1}{\pi W{\left(R_{n}\right)}^{2}}\cdot \iint \exp\left[-\frac{x^{2}+y^{2}}{W{\left(R_{n}\right)}^{2}}\right]\cdot I\left(\frac{W\left(L\right)}{W\left(R_{n}\right)}x,\frac{W\left(L\right)}{W\left(R_{n}\right)}y\right)\rho_{rn}\left(x,y\right)dxdy$$

According to Equations (8) and (10), the distributions of light field and the intensity of echo signal of the *n*-th targets *P*_1*n*_(*x,y*) and *P*_2*n*_ are written as

(11) $$\left\{ \begin{array}{l} P_{1n}\left(x,y\right)=\frac{T_{a}P_{0}}{\tau \sqrt{2\pi }}\cdot \frac{1}{\pi W{\left(R_{n}\right)}^{2}}\exp\left[-\frac{x^{2}+y^{2}}{W{\left(R_{n}\right)}^{2}}\right]\cdot I\left(\frac{W\left(L\right)}{W\left(R_{n}\right)}x,\frac{W\left(L\right)}{W\left(R_{n}\right)}y\right)\\ P_{2n}=\frac{T_{a}{}^{2}T_{0}P_{0}}{\tau_{r}\sqrt{2\pi }}\cdot \iint \frac{1}{\pi W{\left(R_{n}\right)}^{2}}\exp\left[-\frac{x^{2}+y^{2}}{W{\left(R_{n}\right)}^{2}}\right]\cdot I\left(\frac{W\left(L\right)}{W\left(R_{n}\right)}x,\frac{W\left(L\right)}{W\left(R_{n}\right)}y\right)\rho_{rn}\left(x,y\right)dxdy \end{array}\right.$$

Equation (11) shows the theory on 2DGI. Based on Equation (11), 3DGI is obtained by combining all 2DGI on different time slices.

## 3. Simulations and Results

### 3.1. Simulation Setup

**Figure 6:**
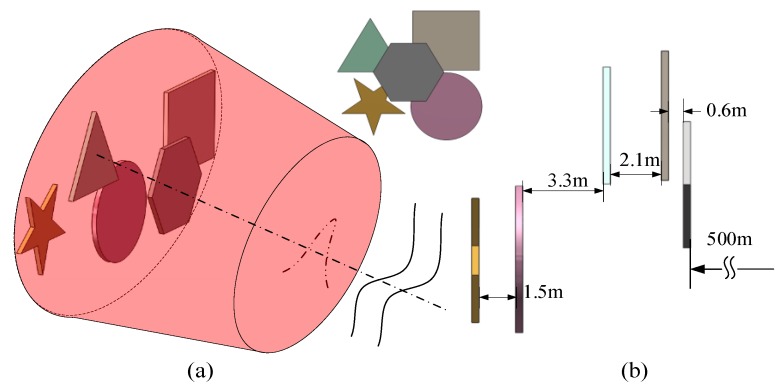
3D target models: (**a**) Perspective view and (**b**) side view.

Numerical simulations are performed based on the above theory to verify the models. As shown in Figure 6, the simulation scene includes a hexagon, square, triangle, circle, and pentagram, and the corresponding feature sizes are 0.16, 0.2, 0.2, 0.2, and 0.07 m, respectively. In addition, the parameters of the pulsed laser are set as follows: Pulse energy is 1 nJ, wavelength is 1550 nm, and the initial pulse width and beam radius are 1 ns and 0.4 m, respectively. The simulations are executed on an Intel Core i5-7600@3.40 GHz computer with 8 GB of RAM memory and Windows 10 as the operating system. The width of the time slice is set to 0.2τ_r_. The noise conforms to Poisson’s distribution. We used the Matlab^®^ built-in function ‘poissrnd(I, n)’ to simulate the environment noise of the echo signal. In the function ‘poissrnd(I, n)’, n is the sampling number which is 400 in the simulation process and I is the mean of the Poisson distribution of random numbers which is 1 × 10^−11^ in the simulation. 

### 3.2. Modeling Verification

**Figure 7:**
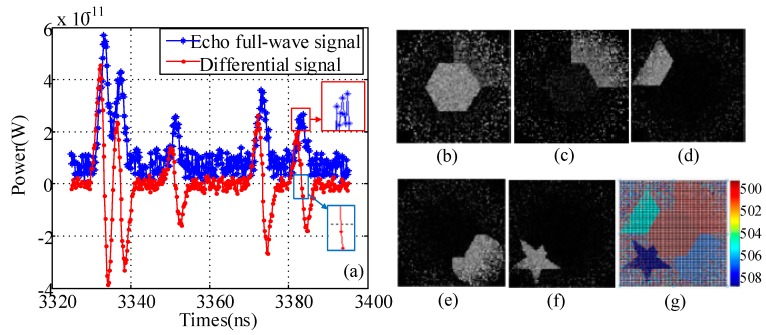
Results of echo signal based on finite difference, 2D, and 3DGI: (**a**) Echo signals based on a single detector and finite difference, (**b**)–(**f**) 2D images under different distances (500–508 m), and (**g**) 3DGI obtained by combining 2DGI. All the above results are without any post-processing.

The 3D images of the targets are constructed with the measurement number at 10,000. The simulation parameters are set as follows: Bandwidth of the receiver is 1 ns, differential distance is 0.06 m, target reflectivity is 0.5, one-way transmission of the laser in the atmosphere is 0.8, and the diameter of the receiver detector is 0.04 m. From the profiles of echo signal based on finite difference, as shown in Figure 7a, five zero crossing are present, indicating the five targets in the scene. Therefore, five time slices are used to obtain 2D images via the corresponding intensity of each target. As shown in Figure 7b–f, each target is clearly constructed. Note that Figure 7b and c includes the hexagon and square information because of the extremely close distance between the two targets. Meanwhile, the peak position fluctuated more than that of the zero crossing, as shown in the rectangular zone, demonstrating that the proposed method is more sensitive than the peak point. By combining all 2D images, we obtain 3DGI (Figure 7g), in which the depth information of each target is obtained by calculating the TOF of each slice.

### 3.3. Comparative Results

**Figure 8:**
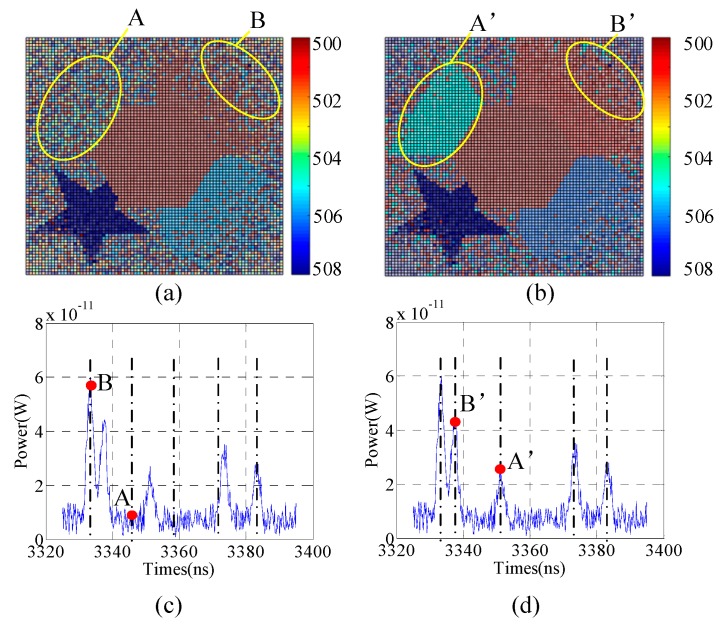
Comparative results: (**a**) And (**b**) are 3DGI based on uniform time-slice method (UTSM) and NUTSM, respectively; (**c**) echo signals based on UTSM, with points A and B representing the corresponding positions of the time slices; and (**d**) echo signals based on NUTSM, with points A’ and B’ representing the corresponding positions of the time slices.

To illustrate the advantages of the proposed method, we compare the simulations from two respects. First, we compare 3DGI based on UTSM and NUTSM. Second, under the condition of a non-uniform time slice, we compare 3DGI based on finite difference and a traditional single detector. In the simulation of comparing 3DGI based on UTSM and NUTSM, the parameters are the same as above. Compared with A (Figure 8a) and A’ (Figure 8b) in 3DGI, the target of the triangle is missing because the triangle is not collected by UTSM. From Figure 8c, point A is not located in the target plane, but point A’ is the target position. Similar comparative results are shown in parts B (Figure 8a) and B’ (Figure 8b), in which the speckle noise in part B is more serious than that in part B’.

**Figure 9:**
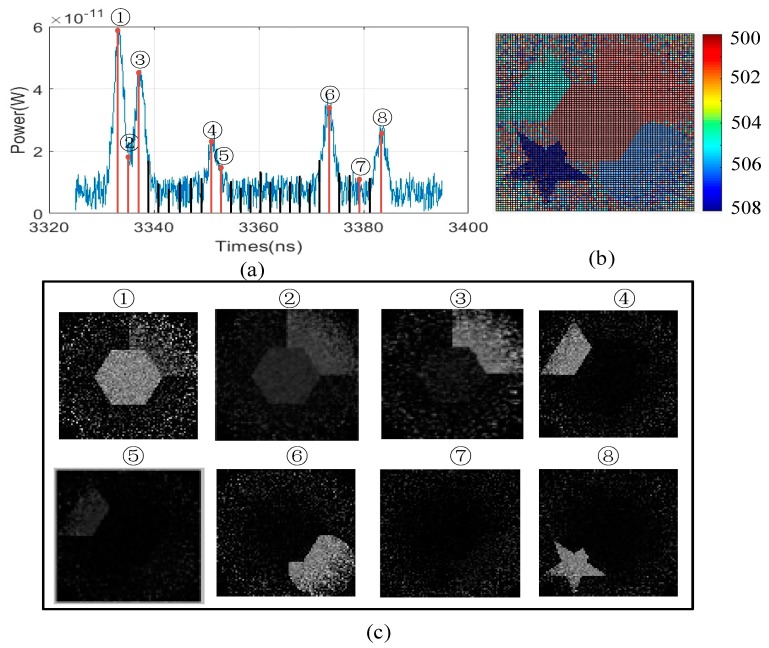
3DGI and 2DGI based on UTSM: (**a**) Echo signal based on a single detector, (**b**) 3DGI via combining 2DGI, and (**c**) corresponding 2D images under different time slices in (**a**).

To obtain the loss information of the target, we also add enough uniform time slices in the traditional 3DGI. The results are shown in Figure 9, in which the time slice is increased from 5 to 29. Therefore, the loss information is obtained in 2DGI, as shown in Figure 9c ④. However, compared with the 3D reconstruction between Figure 9b and Figure 7g, the 3D imaging quality of Figure 7g is better because more speckle noise is introduced by more time slices in the traditional method. Although the loss information of the target is obtained, as shown in Figure 9c ②, ⑤, and ⑦, the performance of the 3D image is sacrificed, an issue that becomes more serious as time slices increase.

**Figure 10:**
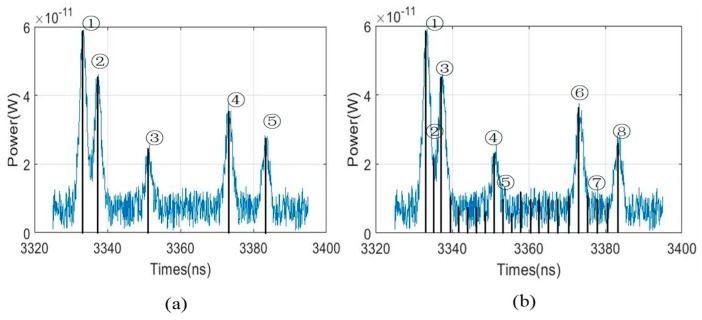

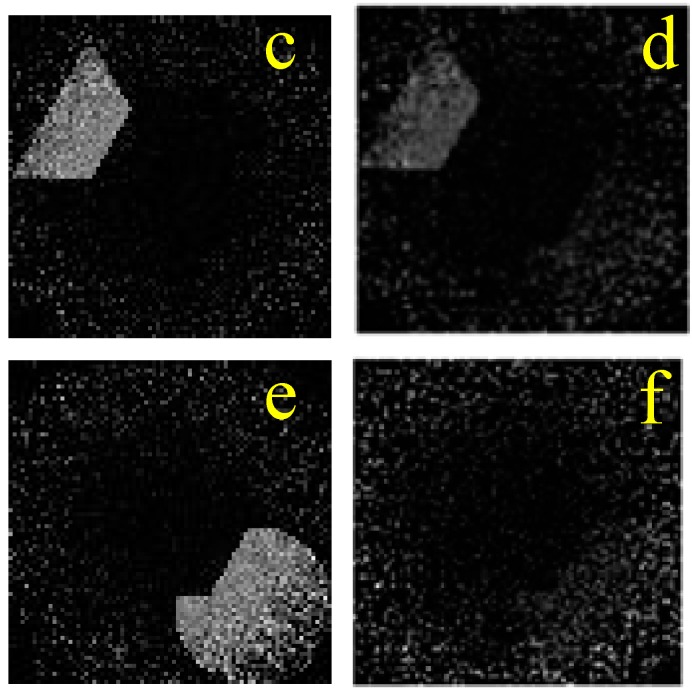
Comparative results based on NUTSM and UTSM. (**a**) is based on NUTSM and (**b**) is based on UTSM. (**c**) And (**e**) are the ghost image under the positions of ③ and ④ corresponding to (**a**). (**d**) And (**f**) are the ghost image under the positions of ⑤ and ⑦ corresponding to (**b**).

Moreover, we compare the quality of two methods under different time slices intuitively. For example, Figure 10c,e are obviously better than Figure 10d,f. Especially for Figure 10f, there is so much speckle noise which affects the performance of the 3DGI. Meanwhile, from the comparative results, we find that more time slices are used in UTSM than NUTSM, which means that more time consumption is cost for UTSM. For example, NUTSM uses 5 time slices, but UTSM uses 8 time slices, which indicates that NTUSM decreases 37.5% of time consumption of GI.

**Figure 11:**
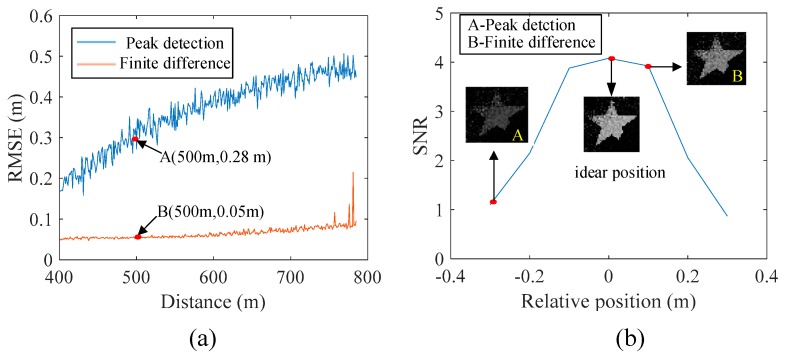
Comparative results based on finite difference and peak detection: (**a**) Root-mean-square errors (RMSEs) based on finite difference and peak detection, and (**b**) Relationship between relative position and signal-to-noise ratio (SNR)

The results of the comparative simulation based on finite difference and peak detection are shown in Figure 11. The parameters of the pulsed laser are set as follows. The pulse energy is 1nJ; the wavelength is 1550 nm; the initial pulse width and beam radius are 1ns and 0.4 m at 500 m. The parameters of the target include that the shape is a pentagram; the feature size is 0.3 m; the reflectivity is 0.5. The transmission rate of one-way of atmosphere is 0.8. We compared the root-mean-square error (RMSE) between the time slice and the target versus the distance of the target based on finite difference and peak detection (Figure 11a), and each RMSE was obtained by 500 measurements at the same distance. The RMSE of finite difference is better than that of peak detection. For example, under the situation that the target distance is at 500 m, the RMSE of peak detection decreases from 0.28 m to 0.05 m, shown in points A and B in Figure 11a. Figure 11b shows the relation of SNR vs. the relative position because we want to further compare the two methods from the aspect of SNR, which is another important index of ghost imaging [6]. The ideal time-slice position is located on the target position, which obtains the highest SNR. However, it is hardly to achieve best SNR by the use of peak detection or finite difference method. Compared with the two methods, the SNR of peak detection is 1.1 (point A of Figure 11b), but finite difference obtains the SNR of 3.98 (point B of Figure 11b). Therefore, the quality of GI increases as the relative position between the time slice and the target decreases, and the method of finite difference can reduce the relative position to improve the quality of the reconstructed image. 

## 4. Discussion

### 4.1. Optimal Differential Distance

From the comparative results above, the performance of 3DGI based on NUTSM combined with finite difference is better than that of the traditional method. The remarkable feature of finite difference which is the key parameter for determining the sensitivity of the zero crossing, results in the relative position error between the time slice and the object. Therefore, the performance of 3DGI versus differential distance should be discussed to obtain the optimal differential distance. Theoretically, the differential signal for a target can be expressed as

(12) $$P_{rd}\left(t\right)=\frac{T_{a}^{2}T_{0}P_{0}\eta_{D}}{\sqrt{2\pi }\tau_{r}}\exp\left[-\frac{1}{2\tau_{r}{}^{2}}{\left(t-\frac{2R+d}{c}\right)}^{2}\right]-\frac{T_{a}^{2}T_{0}P_{0}\eta_{D}}{\sqrt{2\pi }\tau_{r}}\exp\left[-\frac{1}{2\tau_{r}{}^{2}}{\left(t-\frac{2R-d}{c}\right)}^{2}\right]$$

The changing rate of differential signal vs. time is

(13) $$k=\frac{T_{a}^{2}T_{0}P_{0}\eta_{D}}{\sqrt{2\pi }\tau_{r}^{3}}\left\{-\left(t-\frac{2R+d}{c}\right)\exp\left[-\frac{1}{2\tau_{r}{}^{2}}{\left(t-\frac{2R+d}{c}\right)}^{2}\right]+\left(t-\frac{2R-d}{c}\right)\exp\left[-\frac{1}{2\tau_{r}{}^{2}}{\left(t-\frac{2R-d}{c}\right)}^{2}\right]\right\}$$

where *k* is sensitivity of differential signal at time *t*. Specifically, the sensitivity of differential signal at zero crossing is

(14) $$k|{}_{t=\frac{2R}{c}}=\frac{T_{a}^{2}T_{0}P_{0}\eta_{D}}{\sqrt{2\pi }\tau_{r}^{3}}\cdot \frac{2d}{c}\exp\left[-\frac{1}{2\tau_{r}{}^{2}}{\left(\frac{d}{c}\right)}^{2}\right]$$

The changing rate between $k|{}_{t=\frac{2R}{c}}$ and *d* is

(15) $$\frac{d\left(k|{}_{t=\frac{2R}{c}}\right)}{d\left(d\right)}=\frac{T_{a}^{2}T_{0}P_{0}\eta_{D}}{\sqrt{2\pi }\tau_{r}^{3}}\cdot \frac{2}{c}\exp\left[-\frac{1}{2\tau_{r}{}^{2}}{\left(\frac{d}{c}\right)}^{2}\right]-\frac{T_{a}^{2}T_{0}P_{0}\eta_{D}}{\sqrt{2\pi }\tau_{r}^{3}}\cdot \frac{2d}{c}\exp\left[-\frac{1}{2\tau_{r}{}^{2}}{\left(\frac{d}{c}\right)}^{2}\right]\cdot \frac{1}{\tau_{r}{}^{2}c}\left(\frac{d}{c}\right)$$

Let $\frac{d\left(k|{}_{t=\frac{2R}{c}}\right)}{d\left(d\right)}\;=\;0$, we obtain *d = cτ*, which achieves the highest of zero crossing sensitivity.

**Figure 12:**
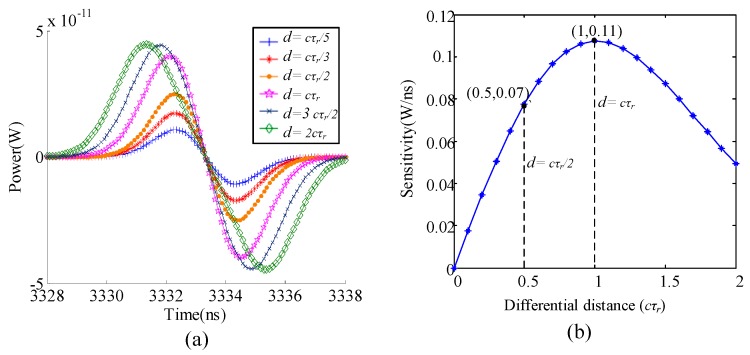
The performance of the time slice under different differential optical paths. (**a**) Echo signal power vs. time. (**b**) Sensitivity vs. differential distance.

Based on the discussion above, we carry out simulations to study the zero crossing sensitivity affected by the differential distance. In Figure 12a, we find that zero crossing sensitivity is variant to different differential distance. According to Figure 12b, *d* = *cτ*_r_ is obtained to achieve the highest zero crossing sensitivity. For example, compared with zero crossing sensitivity at differential distance of *cτ*_r_/2 and *cτ*_r_, zero crossing sensitivity increases from 0.07 to 0.11. 

### 4.2. Issue on Non Zero Crossing

**Figure 13:**
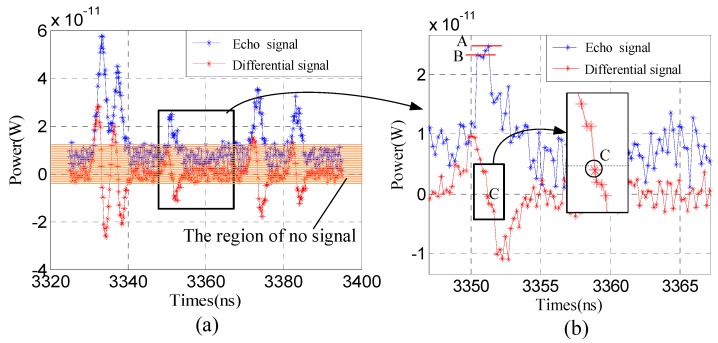
Example of the issue on not exact zero when taking the signal difference. (**a**) The echo signal from the depth of field of view. (**b**) Zooming area for the black rectangle of (**a**).

For the issue on not exact zero when taking the signal difference, actually, zero crossing may be not exact zero rather than a very small value. The key of finite difference is to solve the low accuracy of the peak, and to improve the accuracy of determining target position. In order to clarify this, we carry out simulations based on the parameters of Section 3.1, and the results are shown in Figure 13. In Figure 13a, the blue curve is the echo signal of the depth of field of view, and the five peaks in the scene, the red curve is the difference signal through the finite difference. From the zooming plot (Figure 13b), we find that the crossing point is not zero, but very small. In the region of no signal, there are many fluctuations which means that Poisson random noise affect echo signal based on the finite difference, even the fluctuations in the peak area results in it being difficult to determine which one is the ‘stop’ moment, e.g., points A or B. But there is only one zero crossing, e.g., point C, which is beneficial to obtaining the ‘stop’ moment accurately. Meanwhile, comparing with points A, B and C, the slop of C is obviously larger than A and B, which means the sensitivity of C is higher than A and B, due to the change rate of the echo signal with time at the peak being near zero. Therefore, we can more accurately find the salient target position no matter if the crossing point is zero or not.

## 5. Conclusions

A novel approach based on non-uniform time slice combined with finite difference is proposed to improve the performance of 3DGI. Compared with traditional peak detection combined uniform time-slice method, finite difference obtains differential full-waveform signal, resulting in high sensitivity in the zero crossing and decreasing the effect from background noise. Therefore, the position of the target is more accurate than the traditional peak detection. Meanwhile, NUTSM is suitable for quickly obtaining 3DGI on salient features in the field of view. We studied the relationship between 3DGI performance and the position of the time slice to obtain the optimum differential distance, which achieved the highest zero-crossing sensitivity. Compared with the traditional USTM, NUTSM combined with finite difference shows better performance of 3DGI, which is beneficial for balancing the high resolution and real-time applications.
